# The C-Terminus of H-Ras as a Target for the Covalent Binding of Reactive Compounds Modulating Ras-Dependent Pathways

**DOI:** 10.1371/journal.pone.0015866

**Published:** 2011-01-06

**Authors:** Clara L. Oeste, Beatriz Díez-Dacal, Francesca Bray, Mario García de Lacoba, Beatriz G. de la Torre, David Andreu, Antonio J. Ruiz-Sánchez, Ezequiel Pérez-Inestrosa, Carlota A. García-Domínguez, José M. Rojas, Dolores Pérez-Sala

**Affiliations:** 1 Department of Chemical and Physical Biology, Centro de Investigaciones Biológicas, Consejo Superior de Investigaciones Científicas, Madrid, Spain; 2 Department of Experimental and Health Sciences, Universitat Pompeu Fabra, Barcelona, Spain; 3 Department of Organic Chemistry, Faculty of Sciences, University of Málaga, Málaga, Spain; 4 Unidad de Biología Celular, Área de Biología Celular y del Desarrollo, Centro Nacional de Microbiología, Instituto de Salud Carlos III, Madrid, Spain; Chinese University of Hong Kong, Hong Kong, Special Administrative Region, People's Republic of China

## Abstract

Ras proteins are crucial players in differentiation and oncogenesis and constitute important drug targets. The localization and activity of Ras proteins are highly dependent on posttranslational modifications at their C-termini. In addition to an isoprenylated cysteine, H-Ras, but not other Ras proteins, possesses two cysteine residues (C181 and C184) in the C-terminal hypervariable domain that act as palmitoylation sites in cells. Cyclopentenone prostaglandins (cyPG) are reactive lipidic mediators that covalently bind to H-Ras and activate H-Ras dependent pathways. Dienone cyPG, such as 15-deoxy-Δ^12,14^-PGJ_2_ (15d-PGJ_2_) and Δ^12^-PGJ_2_ selectively bind to the H-Ras hypervariable domain. Here we show that these cyPG bind simultaneously C181 and C184 of H-Ras, thus potentially altering the conformational tendencies of the hypervariable domain. Based on these results, we have explored the capacity of several bifunctional cysteine reactive small molecules to bind to the hypervariable domain of H-Ras proteins. Interestingly, phenylarsine oxide (PAO), a widely used tyrosine phosphatase inhibitor, and dibromobimane, a cross-linking agent used for cysteine mapping, effectively bind H-Ras hypervariable domain. The interaction of PAO with H-Ras takes place in vitro and in cells and blocks modification of H-Ras by 15d-PGJ_2_. Moreover, PAO treatment selectively alters H-Ras membrane partition and the pattern of H-Ras activation in cells, from the plasma membrane to endomembranes. These results identify H-Ras as a novel target for PAO. More importantly, these observations reveal that small molecules or reactive intermediates interacting with spatially vicinal cysteines induce intramolecular cross-linking of H-Ras C-terminus potentially contributing to the modulation of Ras-dependent pathways.

## Introduction

Ras proteins are key regulators of cellular proliferation, survival or senescence. In addition to their role in cellular physiology, Ras proteins are involved in many pathophysiological processes. In fact, mutations in the Ras genes are found in 30% of human cancers [1]. Therefore, since their discovery, Ras oncogenes have been the subject of intense research. The three Ras genes, H-, N-, and K-Ras display extensive homology but also important distinctive features (reviewed in [2], [3]).

Ras proteins are GTP-binding proteins which hydrolyze GTP with the concourse of GTPase activating proteins. Ras activity is controlled by a cycle between the GTP-bound active state and the GDP-bound inactive state. In the GTP-bound state, Ras proteins may interact with effectors including Raf, PI3K and RalGDS, among others [3], [4], to activate various signaling pathways with impact on cell cycle regulation, cytoskeleton organization, transcriptional regulation or membrane trafficking. Ras activity is subjected to a complex modulation at many different levels. Ras activation, requiring the exchange of bound GDP for GTP, can occur by interaction with GTP exchange factors (GEFs), which in turn may transduce signals from growth factor receptors or sense levels of intracellular mediators, including calcium and diacylglycerol. In addition, GDP-GTP exchange can be favoured by the direct action of reactive oxygen or nitrogen species, such as nitric oxide, on Ras proteins [5].

Posttranslational modifications of Ras proteins play a key role in their localization and activity. All Ras proteins are isoprenylated at a cysteine residue present in the CAAX box located at the C-termini [6]. In addition, whereas H-Ras is palmitoylated at two cysteine residues present in the hypervariable domain (C181 and C184), N-Ras contains a single palmitoylation site (C181) and K-Ras4B possesses no cysteine residues but its interaction with cellular membranes is favoured by the presence of a polybasic domain. These differences are critical to determine the traffic and site of action of Ras proteins (reviewed in [4], [7]). Thus, H-Ras is located mainly at the plasma membrane and in endosomes and traffics to the plasma membrane through the conventional secretory pathway. In contrast, K-Ras4B has been reported to interact with microtubules, although the pathway by which it reaches the plasma membrane is not well characterized. In the case of H-Ras, a palmitoylation-depalmitoylation cycle regulates recycling of the protein between the plasma membrane and the Golgi. Moreover, palmitate groups are important for determining the interaction of H-Ras with specific membrane microdomains, its membrane insertion and orientation and its presence in internal membranes, which in turn may influence the engagement of various signaling pathways [8], [9].

Cyclopentenone prostaglandins (cyPG) are reactive lipidic mediators that are generated by dehydration of their parent PG. PGE-type PG give rise to cyPG of the A-series. The J series of cyPG arise from dehydration of PGD_2_ by a sequence of well characterized steps giving rise to the single enone PGJ_2_ and to the dienone PG 15d-PGJ_2_ and Δ^12^-PGJ_2_ [10], [11]. These prostanoids are produced in increased amounts in situations associated with inflammation or oxidative stress and they have been shown to influence cell proliferation and contribute to the resolution of inflammation [12]. cyPG contain an α,β-unsaturated carbonyl group in the cyclopentenone ring. Electrophilic carbons conjugated with this group can suffer the attack of nucleophiles, such as sulphur atoms, and form Michael adducts. Through this mechanism, cyPG may covalently modify proteins, leading to alteration of protein structure and function [13]. We have previously shown that several cyPG may bind to Ras proteins, and that this correlates with Ras activation, as evidenced by an increase in the levels of Ras in its active conformation, and an activation of Ras dependent pathways [14]. Interestingly, we observed that modification and activation of Ras proteins by cyPG with different structure are site-selective. Single enone PG, like PGA_1_ or PGJ_2_ preferentially bound to C118 in H-Ras, which is located in the GTP binding site, whereas cyPG with dienone structure preferentially bound to the C-terminal segment containing C181 and C184 [15]. Interestingly, this preferential binding resulted in the selective modification of the different Ras proteins: while dienone cyPG selectively bound to H-Ras, single enone cyPG could bind to the three Ras proteins, H-, N- and K-Ras4B, because all three possess the C118 residue. In light of these data, we formulated the hypothesis that, dienone cyPG, possessing two electrophilic carbons, could react simultaneously with two different cysteine residues. The fact that H-Ras is the only Ras protein possessing two nearby cysteine residues could constitute the structural basis for its selective modification by cyPG with dienone structure. Moreover, this observation raised the possibility that the C-terminus of H-Ras could be the target for other bifunctional cysteine reagents. Here we have characterized the modification of the C-terminal region of H-Ras by cyPG dienones as well as by other bifunctional cysteine reagents and explored the functional consequences of this modification. Since palmitoylation is a reversible modification, our results suggest that the reactive cysteines present in the H-Ras C-terminus may also be targeted by various small molecules or biological intermediates with reactivity towards vicinal thiols, with diverse consequences on H-Ras localization and function.

## Results

### cyPG with dienone structure bind simultaneously to C181 and C184 of H-Ras C-terminus

cyPG dienones, such as 15d-PGJ_2_ and Δ^12^-PGJ_2_, can covalently modify H-Ras by preferentially binding the C-terminal peptide containing C181 and C184 [15]. Given the bifunctional nature of these dienones, the possibility exists that they simultaneously bind to both cysteine residues. To investigate this point we have used a synthetic peptide corresponding to the tryptic fragment K170-K185 (*m/z* 1662.7) from H-Ras C-terminus.

**Figure 1:**
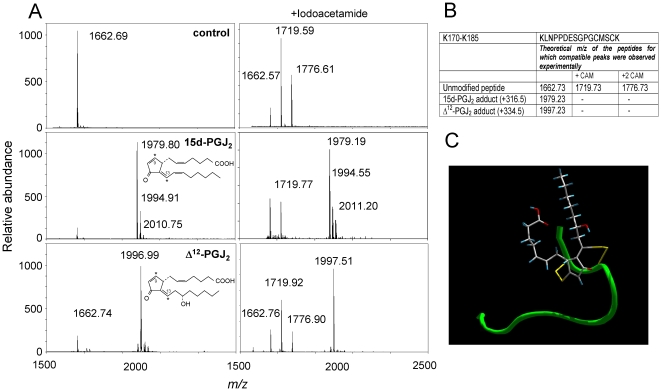
Modification of the K170-K185 peptide from the C-terminal region of H-Ras by cyPG. (A) The synthetic K170-K185 peptide was incubated with the indicated cyPG and the resulting adducts analyzed by MALDI-TOF MS. When indicated, incubation mixtures were treated with 50 mM iodoacetamide after addition of 10 mM DTT. Spectra presented are representative from at least three independent assays per experimental condition. Structures of the cyPG used are shown in insets. Electrophilic carbons are marked by asterisks. (B) Summary of the theoretical peptide adducts for which compatible peaks were observed experimentally. +CAM indicates that the peak is compatible with the formation of carbamidomethyl cysteine subsequent to iodoacetamide treatment. (C) Ribbon diagram for the theoretical backbone structure of the H-Ras K170-K185 peptide (in green) modified by addition of 15d-PGJ_2_ to cysteines 181 and 184. The side-chains of the cysteine residues and the 15d-PGJ_2_ ligand are displayed in yellow and a default atom-type color scheme, respectively.

Incubation of K170-K185 with 15d-PGJ_2_ gave rise to the appearance of a peak of *m/z* 1979.80 corresponding to the addition of one PG molecule (Fig. 1A). Moreover, as previously noted [14], [15], several peaks compatible with the oxidation of the modified peptide (*m/z* 1994.9 and 2010.7) were observed. Reduction and alkylation of the control peptide by subsequent treatment with DTT and iodoacetamide gave the expected peaks (*m/z* 1719.6 and 1776.6) corresponding to the carbamidomethylation of one and two cysteines, respectively. Addition of 15d-PGJ_2_ to the K170-K185 peptide was not reversed by DTT. Interestingly, carbamidomethylation of the 15d-PGJ_2_-modified peptide upon treatment with iodoacetamide was nearly undetectable, suggesting that the 15d-PGJ_2_-modified peptide was basically devoid of free cysteines. Similar results were obtained when the H-Ras C-terminal peptide was modified by Δ^12^-PGJ_2_, another cyPG with dienone structure giving rise to a modified peptide with *m/z* 1997.5. These results, summarized in Fig. 1B, contrast with the behavior of PGJ_2_, a single enone cyPG, which we have previously shown to form one- or two-cyPG adducts (*m/z* 1997.4 and 2331.7, respectively) with the H-Ras C-terminal peptide, thus showing binding to single cysteine residues [15]. Taken together these results indicate that modification of K170-K185 by cyPG dienones may involve intramolecular cross-linking of both C181 and C184. The C-terminal peptide of H-Ras does not show a defined structure in solution [16], but it adopts a precise conformation upon interaction with membranes [17]. Cross-linking between C181 and 184 could impose a more rigid conformation and potentially alter H-Ras membrane interactions. A theoretical molecular model illustrating the feasibility of 15d-PGJ_2_-induced intramolecular cross-linking of the K170-K185 peptide is shown in Fig. 1C.

### The small molecule bifunctional reagent phenylarsine oxide cross-links C181 and C184 of the H-Ras C-terminal peptide

**Figure 2:**
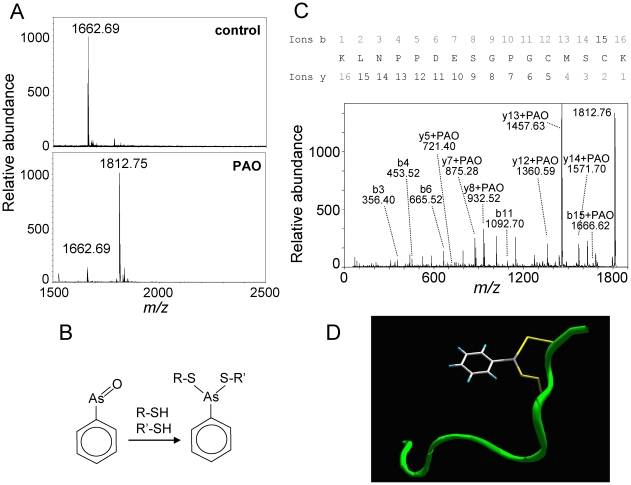
Modification of the K170-K185 peptide by PAO. (A) The K170-K185 peptide was incubated with PAO and analyzed by MALDI-TOF MS. Results are representative from at least four assays. (B) Scheme of the formation of a PAO adduct with two thiol groups. (C) Analysis of the PAO-peptide adduct by MALDI-TOF-TOF MS/MS analysis. The sequence of the peptide with the fragments generated is shown for reference. (D) Ribbon diagram for the theoretical backbone structure of H-Ras K170-K185 peptide model (in green) modified by PAO addition. The side-chains of the cysteine residues and PAO are displayed in yellow and a default atom-type color scheme, respectively.

In view of the ability of cyPG with dienone structure to simultaneously bind the cysteine residues of the H-Ras hypervariable domain, we tested other small bifunctional molecules for cross-linking of these two cysteines. The tyrosine phosphatase inhibitor phenylarsine oxide (PAO, mass 168.02), widely used in signal transduction studies and as an endocytosis inhibitor [18], [19], is also known to form adducts with proteins by reacting with vicinal sulfhydryl groups of proteins forming thioarsine rings [20], [21], [22]. Incubation of K170-K185 with PAO resulted in the formation of an adduct of *m/z* 1812.7 (Fig. 2A), with a mass increment of 150, compatible with the formation of a cross-link between C181 and C184 and one PAO molecule, which implies the loss of the oxygen atom of the PAO molecule plus two hydrogen atoms (Fig. 2B). Analysis of this 1812.7 peak by MALDI-TOF-TOF MS/MS gave fragments compatible with the incorporation of PAO at the proposed sites (Fig. 2C). The smallest fragment detected containing the mass increment compatible with PAO addition corresponded to ion y5 of sequence CMSCK, confirming the site of modification. Figure 2D depicts a homology molecular model of the H-Ras C-terminal peptide after cross-linking elicited by PAO binding.

**Figure 3:**
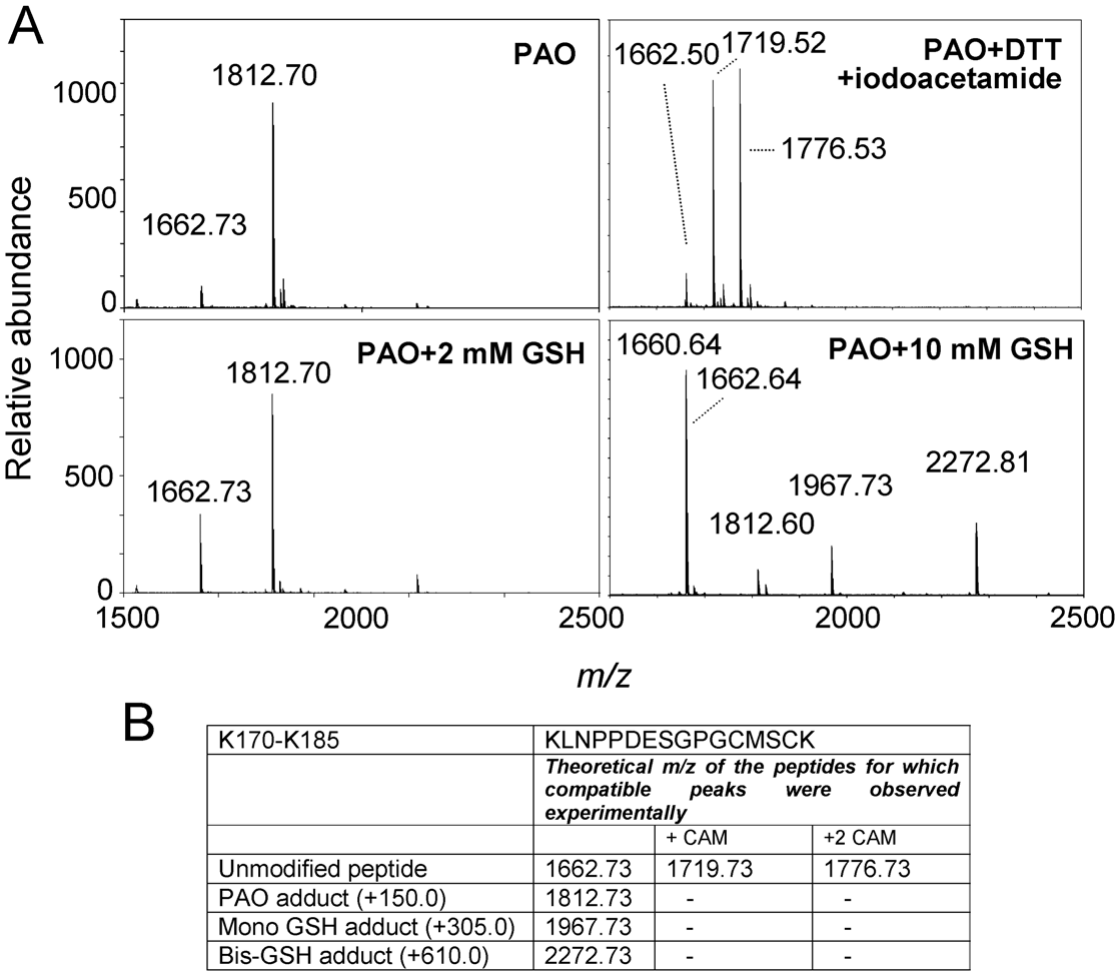
Effect of reducing agents on PAO modification of the K170-K185 peptide. (A) In the upper panels, the K170-K185 peptide was incubated with PAO and analyzed by MALDI-TOF MS. In the right panel, the incubation mixture was treated with 10 mM DTT and subsequently with 50 mM iodoacetamide. In the lower panels, the K170-K185 peptide was incubated with PAO in the presence of the indicated concentrations of GSH before analysis by MALDI-TOF MS. (B) Summary of the theoretical *m/z* of peptides for which compatible peaks were observed experimentally in the assays shown in (A).

In contrast with the cross-linking by cyPG with dienone structure, PAO addition was fully reversible upon treatment with excess DTT. Thus, PAO-modified K170-K185 underwent carbamidomethylation at one or two cysteines after incubation with DTT and iodoacetamide (Fig. 3A, upper panels). Given the reversibility of PAO addition by competing agents like DTT, we were interested in exploring whether PAO-induced modification of the K170-K185 H-Ras peptide would occur in the presence of a biologically relevant thiol compound at concentrations close to those encountered in vivo. Interestingly, the presence of glutathione (GSH) in the incubation mixture reduced the proportion of PAO-peptide adduct formed in a concentration-dependent manner, as it can be deduced from the abundance of modified (*m/z* 1812.7) vs parent (*m/z* 1662.7) peptide (Fig. 3A, lower panels). However, a substantial proportion of PAO-peptide adduct could still be detected at GSH concentrations of up to 10 mM, thus suggesting that H-Ras modification by PAO could occur in cells. Remarkably, at 10 mM GSH, peaks of *m/z* 1967. 7 and 2272.8, compatible with the mono and di-glutathiolation of K170-K185 were also observed. A summary of the species encountered in the MS analysis of the K170-K185 peptide adducts after treatment with the various agents used is shown in Fig. 3B.

### PAO binds to H-Ras C-terminus in vitro and in intact cells

**Figure 4:**
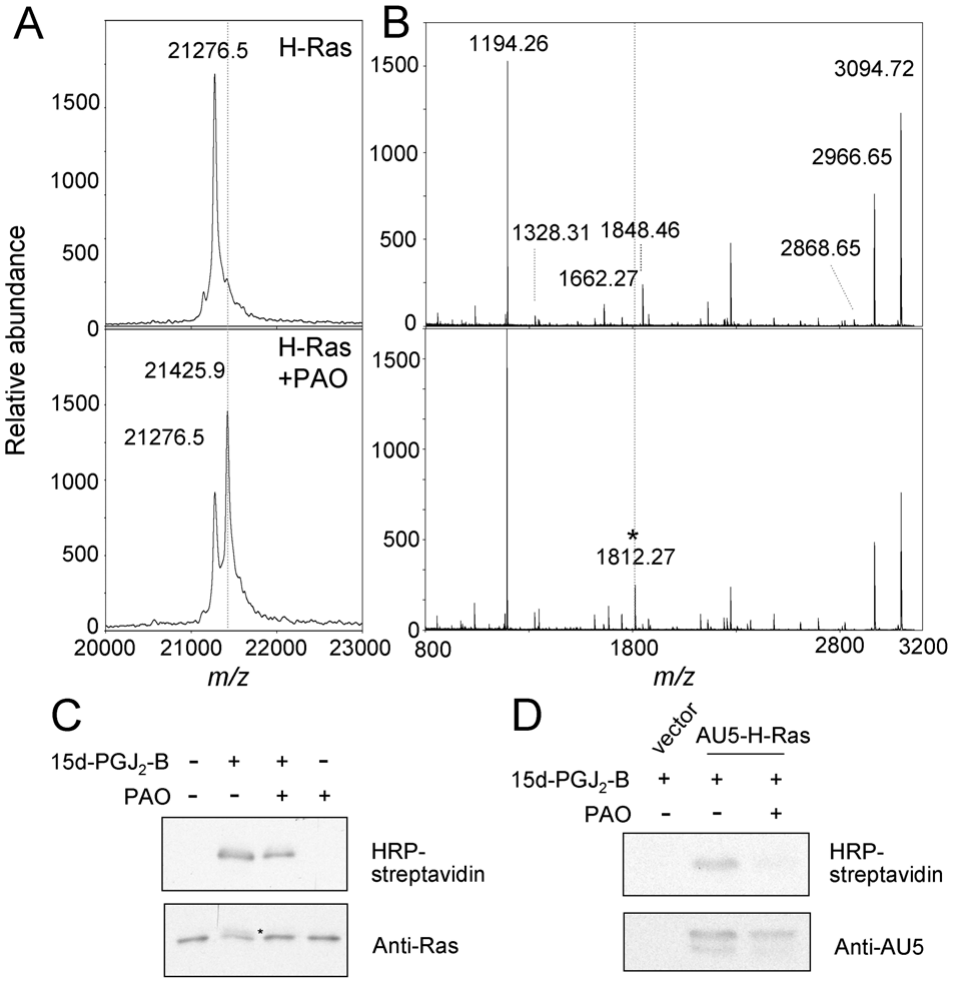
PAO binds to the C-terminal peptide of H-Ras in vitro and in cells. (A) Full length H-Ras was incubated with PAO and analyzed by MALDI-TOF MS. (B) Control or PAO-treated H-Ras was subjected to tryptic digestion and the resulting peptides analyzed by MALDI-TOF MS. The peak of *m/z* 1812.27, corresponding to the formation of an adduct between PAO and the K170-K185 peptide, is marked by an asterisk. Dotted lines mark the positions equivalent to those of the modified species in the untreated samples. (C) H-Ras at 5 µM was incubated with 50 µM PAO for 30 min before addition of 1 µM biotinylated 15d-PGJ_2_ (15d-PGJ_2_-B) for 1 h. (D) COS-7 cells were transiently transfected with empty vector or a plasmid coding for AU5-H-Ras. 24 h after transfection cells were pre-treated with vehicle or 1 µM PAO for 90 min before incubation with 5 µM 15d-PGJ_2_-B for 90 min. Incubation mixtures from (C) and cell lysates from (D) were analysed by SDS-PAGE followed by transfer to membrane and detection of incorporated biotin with horseradish peroxidase (HRP)-streptavidin and of the Ras protein by immunoblot with anti-pan Ras or anti-AU5 antibody and enhanced chemiluminescence (ECL). Results shown are representative of at least three assays.

In view of the above results we explored whether PAO could bind to the full length H-Ras protein in vitro, and if so, whether the peptide containing C181 and C184 was the preferred site for interaction. As it can be observed in Fig. 4A incubation of full-length H-Ras with PAO resulted in the formation of an adduct containing one PAO molecule bound to H-Ras, as clearly detected by MALDI-TOF MS analysis. Moreover, tryptic digestion of the modified protein revealed the appearance of a peak with *m/z* 1812.7 as the main modified peptide, which corresponded to the addition of one PAO molecule to the K170-K185 tryptic peptide (Fig. 4B). We have previously reported that biotinylated 15d-PGJ_2_ (15d-PGJ_2_-B) binds to H-Ras in vitro and in intact cells at the same peptide [14], [15]. Therefore, we explored the capacity of PAO to block the binding of 15d-PGJ_2_-B to H-Ras, by using a gel-based assay. Pre-incubation with PAO clearly reduced the incorporation of 15d-PGJ_2_-B into H-Ras in vitro (Fig. 4C). This could be evidenced both by the reduction of the H-Ras-associated biotin signal (28%±7 reduction with respect to vehicle-treated Ras, mean ± standard deviation of three assays), and by the reduction of the amount of 15d-PGJ_2_-B modified protein, which appears as a higher molecular weight band (marked by an asterisk), as we have previously described [15]. We next explored the effect of PAO on 15d-PGJ_2_-B binding to H-Ras in intact cells by using COS-7 cells transiently transfected with an expression vector for AU5-tagged H-Ras. Consistent with previous reports, AU5-H-Ras appeared in cell lysates as a doublet, reflecting different degrees of posttranslational processing, with the lower band corresponding to the fully processed H-Ras protein [6]. Incubation with PAO markedly reduced the extent of modification of H-Ras by 15d-PGJ_2_-B in COS-7 cells (Fig. 4D). Quantification of the biotin signal corrected by AU5-H-Ras protein levels yielded a 55%±6 reduction (mean ± standard deviation of three experiments) in 15d-PGJ_2_-B incorporation into H-Ras in PAO pre-treated cells. These results indicate that PAO binds to the C-terminus of H-Ras in intact cells.

### Functional consequences of H-Ras modification by PAO

**Figure 5:**
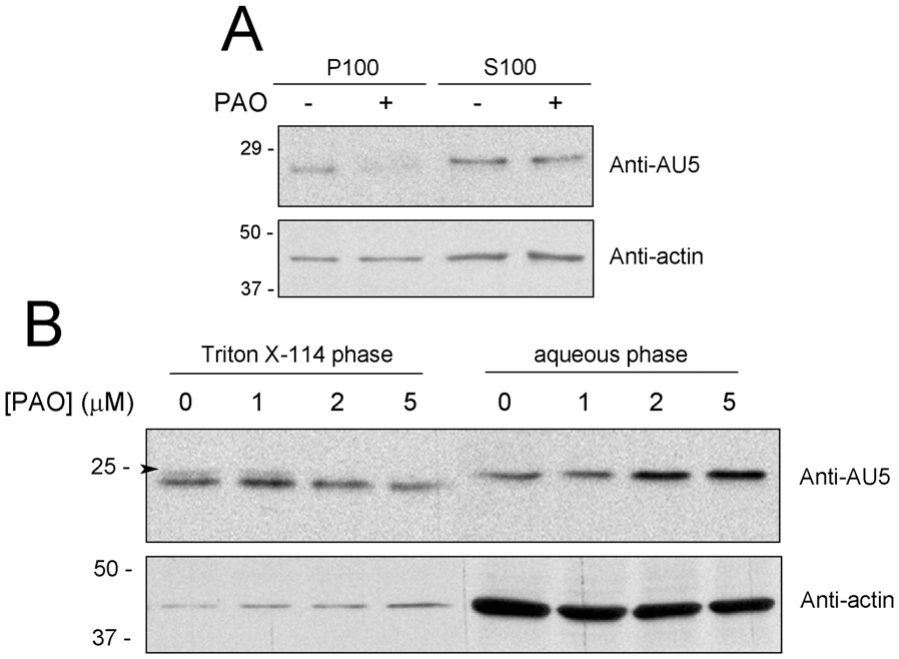
Effect of PAO on H-Ras subcellular localization. (A) COS-7 cells were transiently transfected with expression vectors coding for AU5-H-Ras wt. After serum starvation for 16 h, cells were treated with vehicle or 1 µM PAO for 1 h. Cells were lysed and postnuclear supernatants were separated into S100 and P100 fractions by ultracentrifugation at 200,000× g for 30 min. (B) AU5-H-Ras transfected cells were treated with the indicated concentrations of PAO for 90 min and cell lysates were subjected to fractionation in Triton-X114. The amount of AU5-H-Ras present in the various fractions was estimated by western blot with anti-AU5 antibody (upper panels). The upper component of the H-Ras doublet is indicated by an arrowhead. Levels of actin were assessed as control (lower panels). Blots shown are representative of three assays.

**Figure 6:**
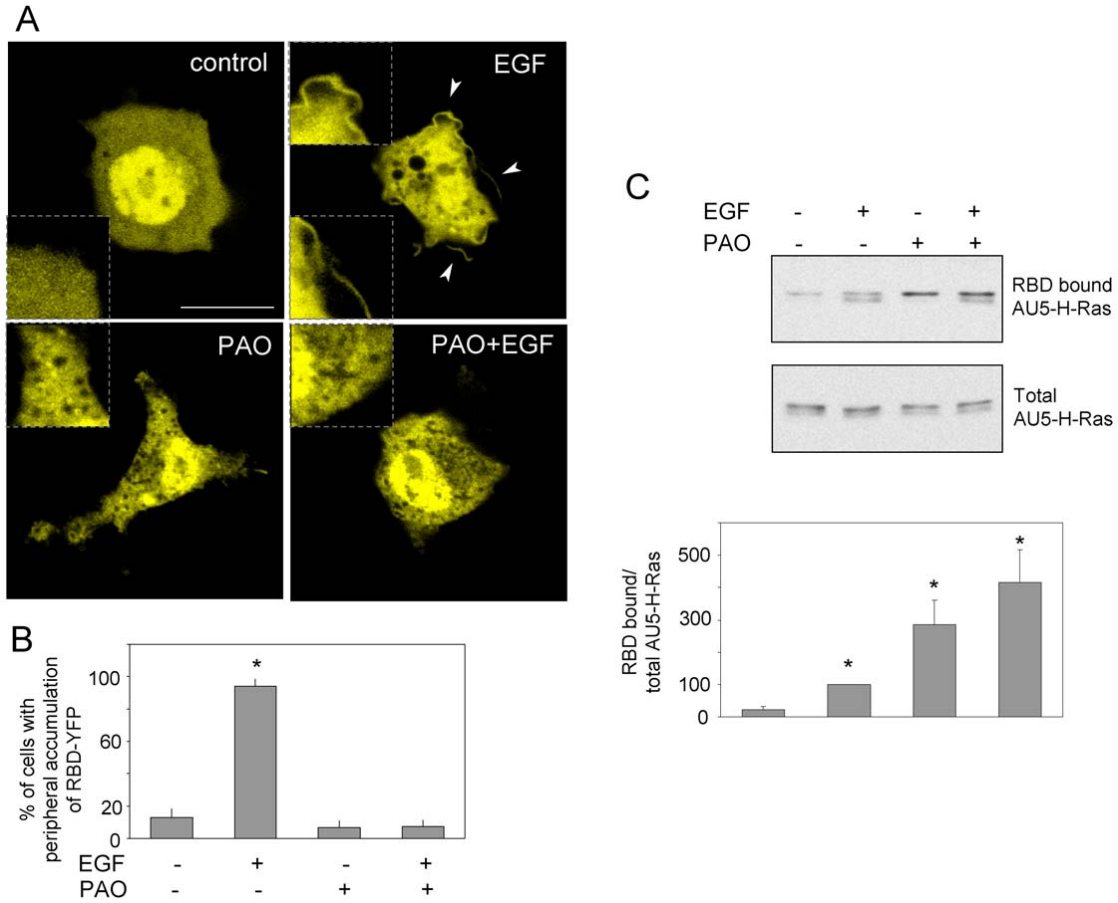
Effect of PAO on Ras activation. (A) COS-7 cells were transfected with YFP-RBD, serum starved for 16 h and pre-incubated with vehicle or 1 µM PAO for 1 h before stimulation with 100 nM EGF. Cells were visualized live by confocal fluorescence microscopy. Shown are representative images from at least five experiments with similar results. Dotted inserts show enlarged areas from the same cells for better visualization. Arrowheads mark the accumulation of YFP-RBD at defined locations of the cell periphery. Bar, 20 µm. At least 40 cells were monitored per experimental condition and the percentage of cells showing accumulations of YFP-RBD at the cell periphery is shown in (B) as average values ± standard error of mean (SEM) of five independent experiments. (C) Cells were treated as above and cell lysates were subjected to pull-down on GST-RBD beads for collection of Ras in its active conformation. Levels of RBD-bound and total AU5-H-Ras were assessed by western blot. Results shown are representative of three assays. The lower panel presents the quantitation of RBD-bound AU5-H-Ras/total AU5-H-Ras ratios from three independent experiments as average values ± SEM. *p<0.05 vs vehicle.

Binding of dienone cyPG or of PAO, as displayed in the theoretical models, would likely alter the conformational tendencies, and, more importantly, the chemical nature of H-Ras C-terminus. In addition, since the hypervariable domain cysteine residues are sites of palmitoylation, incorporation of PAO could have consequences for the membrane binding or the hydrophobicity of the modified protein. Therefore, we explored whether treatment of cells with PAO led to alterations in H-Ras subcellular distribution. Treatment of AU5-H-Ras transfected COS-7 cells with PAO led to a decrease in the amount of membrane associated H-Ras, as evidenced by cell fractionation into P100 and S100 fractions (Fig. 5A). Partition into the detergent Triton-X114 has been shown to constitute an assay for protein hydrophobicity [23] and it has been used as an index of H-Ras lipidation [24]. Indeed, isoprenylation has been shown to increase the proportion of H-Ras partitioning into the Triton-X114 phase, this being further increased by palmitoylation [6]. A Triton X-114 partition assay showed that treatment of AU5-H-Ras transfected cells with PAO reduced the amount of H-Ras protein in the detergent phase in a concentration-dependent manner, mostly at the expense of the upper component of the AU5-H-Ras doublet, with a concomitant increase in the protein recovered in the aqueous fraction (Fig. 5B). Therefore, these results suggest that PAO impairs lipidation of H-Ras in cells. We were then interested in assessing the effect of PAO on H-Ras function. Given the reversible nature of PAO binding we employed a Raf Ras binding domain (Raf-RBD) redistribution assay, which allows exploring Ras activation in intact cells. The YFP-RBD construct showed a diffuse distribution in transiently transfected COS-7 cells. Treatment with EGF led to a more irregular intracellular YFP-RBD pattern and, above all, to the appearance of defined accumulations of the fluorescent construct at the cell periphery, in some cases at the edge of membrane ruffle-like structures, thus indicating the recruitment of YFP-RBD by activated Ras at the plasma membrane (Fig. 6A). Incubation with PAO caused intracellular redistribution of YFP-RBD, resulting in a more particulate pattern. Interestingly, pre-treatment with PAO completely blocked EGF-induced recruitment of YFP-RBD to the plasma membrane. This indicates that Ras activation at the plasma membrane is blocked by PAO. Quantitation of these results is shown in Fig. 6B.

We next explored Ras activation by means of a RBD pull-down assay (Fig. 6C). EGF clearly activated H-Ras in this assay, as deduced from the increase in the retention of AU5-H-Ras on RBD beads. No AU5 signal was detected in the pull-down from cells transfected with empty vector (results not shown). Notably, treatment with PAO alone induced the retention on RBD beads of the slower migrating AU5-H-Ras band and did not reduce EGF-induced Ras activation in this assay. Taken together these results suggest that PAO treatment targets H-Ras in intact cells and alters H-Ras-mediated signaling by selectively blocking EGF-mediated Ras activation at the cell membrane and potentially inducing Ras-Raf interaction at other locations.

### The cysteine cross-linking agent dibromobimane targets H-Ras hypervariable domain

**Figure 7:**
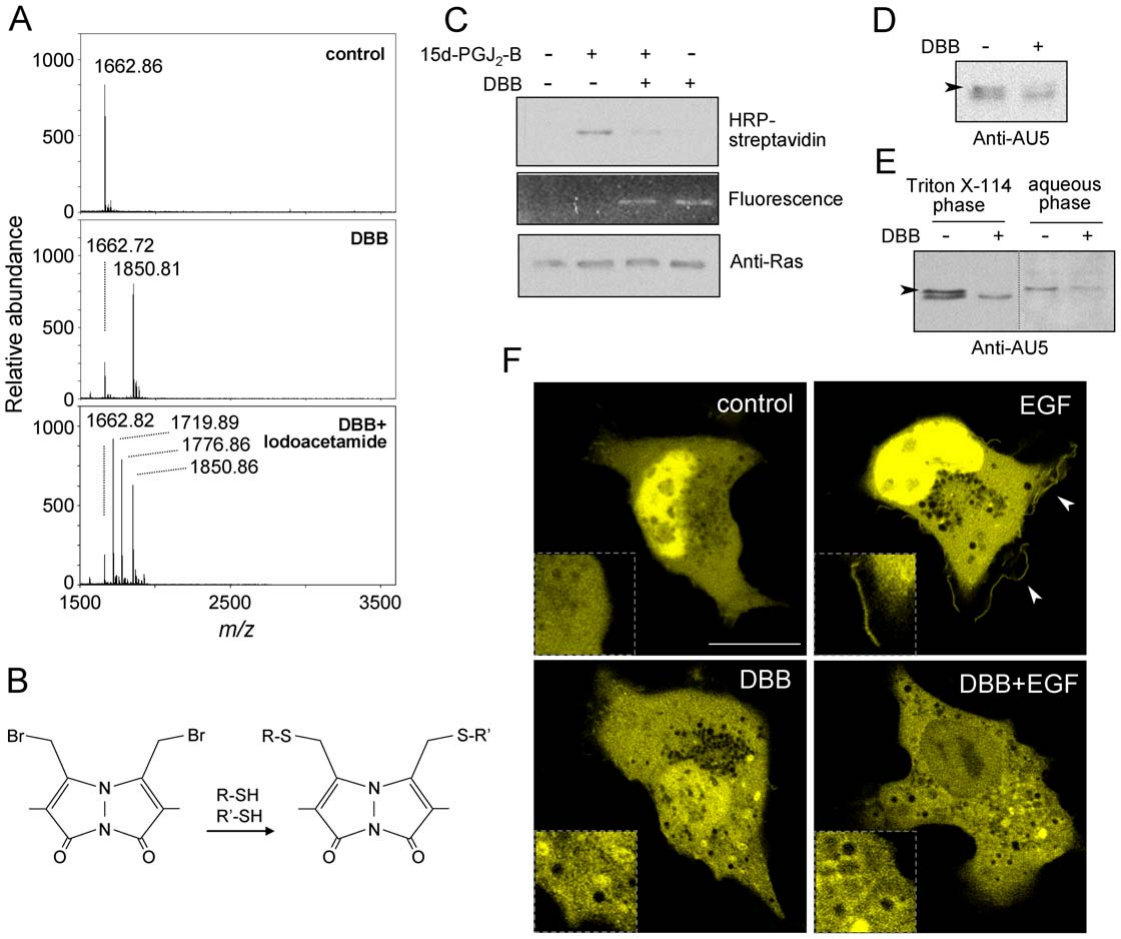
Interaction of the bifunctional reagent DBB with H-Ras. (A) Modification of the K170-K185 peptide by DBB. The K170-K185 peptide was incubated with DBB, as described in the Methods section. When indicated the incubation mixture was subjected to reduction and alkylation with iodoacetamide to modify free cysteines. Results are representative of three independent assays. (B) Scheme of the cross-linking of two thiol groups by DBB. (C) Competition of 15d-PGJ_2_-B binding to H-Ras by DBB. H-Ras at 5 µM was incubated with 50 µM DBB for 30 min before addition of 1 µM 15d-PGJ_2_-B for 1 h. Incorporation of the biotinylated PG was assessed by SDS-PAGE, protein blot and detection with horseradish peroxidase (HRP)-streptavidin (upper panel). Incorporation of DBB (middle panel) and total H-Ras levels (lower panel) were assessed by fluorescence detection and western blot with anti-pan Ras antibody, respectively. (D) COS-7 cells transfected with the AU5-H-Ras vector were treated in the absence or presence of 50 µM DBB for 1 h. Aliquots from total cell lysates (10 µg of protein) were analyzed by SDS-PAGE. The upper component of the AU5-H-Ras doublet is indicated by an arrowhead. (E) COS-7 cells transfected with the AU5-H-Ras vector were treated with DBB and subjected to fractionation in Triton-X114 as above. In (D) and (E) levels of AU5-H-Ras were assessed by western blot with anti-AU5 antibody. The dotted line marks where lanes from the same gel have been cropped. (F) COS-7 cells transfected with YFP-RBD as above were treated with 50 µM DBB for 1 h before stimulation with 100 nM EGF, as indicated, and visualized live by confocal fluorescence microscopy. Dotted inserts show enlarged areas from the same cells for better visualization. Arrowheads mark the accumulation of YFP-RBD at defined locations of the cell periphery. Bar, 20 µm. Shown are representative images from three experiments with similar results.

The results shown above suggest that small molecules interacting with spatially vicinal cysteines may induce an intramolecular cross-linking of H-Ras hypervariable domain and alter Ras distribution and signaling. To further substantiate this point, we studied the effect of dibromobimane (DBB, mass 350.01), a reagent known to cross-link cysteine residues within 3–6 Å distance [25]. DBB was shown to bind K170-K185 by MS analysis (Fig. 7A). The *m/z* of the modified peptide (1850.81) was compatible with addition of one DBB molecule to C181 and C184, according to the reaction depicted in Fig. 7B (expected mass increment 188.0). Formation of peptide dimers was not detected, again suggesting the occurrence of intramolecular cysteine cross-linking by DBB. Moreover, reduction and alkylation with iodoacetamide did not result in carbamidomethylation of the DBB-modified peptide, thus ruling out the presence of free cysteines. As observed for PAO, treatment of full length H-Ras with DBB reduced the modification of the protein by 15d-PGJ_2_-B, suggesting that DBB binds to H-Ras at the C-terminal peptide (Fig. 7C). Indeed, direct binding of DBB to H-Ras could be evidenced by fluorescence detection of the gels, taking advantage of the fluorogenic properties of this compound upon replacement of both bromines [25] (Fig. 7C). Although DBB has been widely used in cysteine mapping studies in vitro, its effects in cultured cells have not been explored. Therefore, at this point we went on to assess whether DBB recapitulated some of the effects of PAO in intact cells. A concentration of 50 µM DBB was required to detect binding of this compound to intracellular components, as observed by confocal fluorescence microscopy (results not shown). This concentration did not affect cell viability nor elicited morphological changes. Under these conditions, treatment of AU5-H-Ras-transfected cells with DBB reduced the levels of the upper component of AU5-H-Ras doublet (Fig. 7D) and led to the virtual disappearance of this band from the detergent phase in the Triton X-114 partition assay (Fig. 7E). Finally, using the Raf-RBD redistribution assay, we observed that in the presence of DBB, YFP-RBD adopted a particulate intracellular pattern and EGF failed to induce the translocation the YFP-RBD construct to the cell periphery or to membrane ruffle-like structures (Fig. 7F). Therefore, DBB markedly alters H-Ras homeostasis and function.

## Discussion

Ras proteins are critical signaling molecules that integrate information from growth factor receptors and intracellular mediators. Ras proteins may sense changes in calcium concentration or redox state and engage various signaling pathways leading to the regulation of basic cellular functions. The ability of Ras proteins to act as sensors of various stimuli in different cell compartments relies in part in the complex array of posttranslational modifications they can undergo. Ras proteins may be subjected to isoprenylation and accompanying modifications, proteolysis and carboxyl methylation, phosphorylation, palmitoylation, ubiquitination and various non-enzymatic modifications including nitrosylation, thiolation or addition of lipid electrophiles [14], [26], [27], [28]. Here we show that the C-terminal end of H-Ras proteins may be the target for both endogenous reactive molecules and exogenous chemicals that cross-link nearby cysteines, thus opening new possibilities for the regulation of Ras proteins and potentially for H-Ras-selective signaling.

In this and previous works we have reported that Ras proteins are targets for modification by endogenous electrophilic eicosanoids, like cyPG and isoprostanes [14], [15]. From a structural point of view, different patterns of modification by eicosanoids with different structure were encountered, with single enone compounds preferentially modifying C118, which is present in all three Ras proteins, and dienone compounds, like 15d-PGJ_2_, binding preferentially to the C-terminal peptide containing C181 and 184, present only in H-Ras. Analogously to the selective modification and activation of H-Ras, but not N-Ras or K-Ras4B, by 15d-PGJ_2_ [15], we have observed that PAO, shown here to modify H-Ras, did not affect the electrophoretical mobility or activation of AU5-K-Ras4B (results not shown). This suggests that the presence of two close cysteine residues in the hypervariable domain of H-Ras may be the main determinant for its selective modification by cyPG dienones, and possibly by other bifunctional cysteine reagents.

In this context, various recent evidences suggest that cyPG dienones could preferentially modify proteins containing cysteines in close proximity. Given the stability of this modification, this could result in intra- or inter-molecular cross-linking. Indeed, we have recently reported that dienone cyPG, like 15d-PGJ_2_ and Δ^12^-PGJ_2_ induce cross-linking of the P1-1 isoform of glutathione S-transferase (GST), which is involved in cancer chemoresistance, through a mechanism likely involving cysteine residues present in different GSTP1-1 monomers [29]. Moreover, 15d-PGJ_2_ has also been shown to directly cross-link c-Jun monomers in vitro [30]. In view of these findings it would be interesting to explore whether proteins with neighboring cysteines important for function, such as thioredoxins, tyrosine phosphatases, Rho proteins or Hsp90 constitute preferential targets for cyPG with dienone structure [20], [31], [32].

It should be taken into account that the cysteine residues of H-Ras targeted by the agents used in this study are the palmitoylation cysteines located in the hypervariable domain. Since H-Ras palmitoylation plays a key role in subcellular localization [8], modification of these cysteine residues could be expected to alter H-Ras distribution. We previously observed that treatment of cells with 15d-PGJ_2_ did not disrupt membrane association of H-Ras, although an increased extractability in detergent was noted, indicative of potential changes in H-Ras membrane microdomain distribution [15]. The results presented here indicate that C181 and C184 of H-Ras can be targeted by several bifunctional molecules of diverse structure, such as PAO and DBB. Our results indicate that H-Ras modification by these agents takes place in vitro and in cells. Moreover, treatment with PAO decreases H-Ras hydrophobicity and membrane binding. In turn, the most obvious effect of DBB under the conditions studied is a reduction in the levels of the upper component of the H-Ras doublet, putatively the non-palmitoylated form, causing its disappearance from the detergent fraction. A reduction in the levels of the slower migrating form of H-Ras was also observed with high (10 µM) PAO concentrations (results not shown). Whether the decrease in the detection of the upper H-Ras band is due to protein aggregation, degradation or other mechanisms, it will be the subject of further studies.

Palmitoylation is a reversible modification, with a half-life that has been measured as approximately 20 min compared to the 20 h half-life of the protein [33]. In addition, Ras depalmitoylation can be stimulated by Ras activation [4], whereas S-nitrosocysteine treatment has been reported to modulate palmitate turnover by stimulating H-Ras deacylation and allowing subsequent reacylation [34]. It is possible therefore, that during the palmitoylation-depalmitoylation cycle of H-Ras, C181 and C184 may become available for chemical modification by various agents or by oxidation. Depending on its stability, this modification could interfere with subsequent palmitoylation and palmitoylation-dependent subcellular localization or microdomain partitioning of H-Ras. According to the current knowledge, depalmitoylation of H-Ras would occur subsequently to activation and allow recycling of the protein towards the Golgi complex, where it would be re-palmitoylated. Indeed, non-palmitoylatable versions of H-Ras have been found to localize primarily on the Golgi and signal mainly from this platform in response to growth factor stimulation [8]. In addition, active H-Ras has been detected at the endoplasmic reticulum and on recycling endosomes in route towards the plasma membrane [35]. Interestingly, mutation of C184 for a more hydrophobic leucine residue has been proposed to be able to substitute for the presence of palmitate at this position and support binding of H-Ras to plasma membrane and recycling endosomes [35]. In light of our data, it would be tempting to hypothesize that the modification of C181 and C184 of H-Ras through the addition of electrophilic endogenous mediators or chemical agents could differentially affect the site of action of this GTPase depending on the structure and/or hydrophobicity of the bound molecule.

We have previously shown that modification of Ras proteins by various eicosanoids, including 15d-PGJ_2_, PGA_1_ [15], Δ^12^-PGJ_2_ or 8-iso-PGA_1_ (Oeste et al., unpublished results), correlates with activation of Ras-dependent pathways, although the precise site of H-Ras activation by these various eicosanoids needs to be studied in detail. Here we have observed that PAO induces an increase in H-Ras binding to RBD. Moreover, the observations that both PAO and DBB induce a cytosolic particulate pattern of YFP-RBD in cells could be indicative of the activation of Ras proteins in endomembranes. Although further work is needed to establish this point, our results with PAO selectively blocking redistribution of YFP-RBD towards the plasma membrane (live cell assay), but not the total RBD binding capacity of H-Ras after EGF stimulation (pull-down assay) would support this hypothesis. Moreover, we observed retention of the slower migrating form of H-Ras on RBD beads upon treatment with PAO. Whether this band represents PAO-modified protein and its precise subcellular localization will be the subject of future studies.

In this work we have used various electrophilic agents as tools to target the palmitoylation cysteines of H-Ras. However, it should be kept in mind that the compounds used may bind to multiple cellular targets. Moreover, thiol-reactive compounds may alter the cellular redox state and have an impact on the oxidative modifications of proteins [21], [36]. In particular, PAO, widely used as a tyrosine phosphatase inhibitor in cells, may interfere with numerous signaling pathways [20], [21]. Indeed, interaction of PAO with Ras signaling pathways has been reported to lead to diverse effects, in some cases of complex interpretation. PAO has been reported to block Ras activation by insulin in murine fibroblasts [37], but not to reduce EGF-induced Ras activation in HEK-293 cells [38]. Our results showing that H-Ras proteins can be direct targets for modification by this compound add further complexity to this picture and call attention to the need of interpreting these results cautiously.

In summary, our results show that bifunctional cysteine reagents can target the hypervariable region of H-Ras and that this interaction may have multifaceted consequences for Ras distribution and signaling.

## Materials and Methods

### Materials

The prostanoids used throughout this study were from Cayman Chemical (Ann Arbor, MI). Human recombinant H-Ras was from Calbiochem-Novabiochem (San Diego, CA). Anti-AU5 monoclonal antibody was from Berkeley Antibody Company (Berkeley, CA), anti-pan Ras (Ab-3) was from Merck. Phenylarsine oxide (PAO), dibromobimane (DBB) and epidermal growth factor (EGF) were purchased from Sigma-Aldrich (St. Louis, MO). Raf-Ras binding domain (Raf-RBD) protein agarose beads were from Cytoskeleton, Inc.

### Synthesis of H-Ras C-terminal peptide

Peptide KLNPPDESGPGCMSCK, corresponding to amino acids K170 to K185 of human H-Ras, henceforth referred to as K170-K185, was chosen as a model because it is the major modified peptide detected after tryptic digestion of 15d-PGJ_2_- and Δ^12^-PGJ_2_-treated H-Ras [15]. The peptide was produced by solid phase methods [39] in an ABI 433A synthesizer (Applied Biosystems, Foster City, CA), using Fmoc (9-fluorenylmethoxy carbonyl) chemistry and 0.1-mmol-scale FastMoc protocols. After chain assembly, it was cleaved from the resin by acidolysis with trifluoroacetic acid/water/ethanedithiol/triisopropylsilane (94∶2.5∶2.5∶1 v/v, 90 min, 25°C), precipitated with chilled methyl tert-butyl ether, redissolved in 10% (v/v) acetic acid and lyophilized. Purification by preparative HPLC (Phenomenex C_18_ column, 21.2×250 mm, 10 µm particle size, linear gradient of solvent B into A –solvent A, 0.045% TFA in H_2_O; solvent B, 0.036% in acetonitrile– over 35 min, 25 ml/min flow rate) yielded fractions containing the target dithiol peptide in highly pure form, with a mass of 1662.73 by MALDI-TOF mass spectrometry.

### Plasmids and transfections

The plasmids pCEFL-KZ-AU5 and pCEFL-KZ-AU5-H-Ras wt have been previously described [14]. An expression plasmid containing the Ras-binding domain of cRaf (RBD) fused to the Yellow Fluorescent Protein (pEYFP-RBD) was prepared from a RBD-pECFP plasmid generously donated by Dr. MR Philips. COS-7 cells were grown in DMEM supplemented with 10% fetal bovine serum, 2 mM glutamine, 100 U/ml penicillin and 100 µg/ml streptomycin. Transient transfections were performed using Lipofectamine 2000 (Invitrogen), according to the instructions of the manufacturer. Treatments with the various agents used were performed in the absence of serum. Prostanoids and PAO were added in DMSO (vehicle) and control cells received an equivalent amount of vehicle.

### Analysis of the interaction between H-Ras and small molecules in vitro

H-Ras or K170-K185 Ras peptide were incubated at 5 µM in the presence of prostanoids, DBB or PAO at 50 µM or vehicle (DMSO), as previously described [15]. For some experiments, H-Ras incubation mixtures were then subjected to digestion with trypsin for 4 h at 37°C. When indicated, aliquots of H-Ras, tryptic digests or K170-K185 peptide were subsequently incubated with 10 mM DTT for 30 min followed by 50 mM iodoacetamide for 30 min at r.t. before purification on ZipTip C18 (Millipore) and MALDI-TOF MS analysis on an AUTOFLEX III MALDI-TOF-TOF instrument (Bruker-Franzen Analytik, Bremen, FRG) operated in the positive mode, as reported in detail [15]. Selected peptides were further analyzed by MALDI-TOF-TOF MS/MS.

Interaction of 15d-PGJ_2_-B and recombinant H-Ras was also explored by SDS-PAGE, protein transfer to Immobilon-P membranes and detection of incorporated biotin with horseradish peroxidase (HRP)-Streptavidin and enhanced chemiluminescence (ECL), as previously described [14]. Direct binding of DBB to H-Ras was assessed by visualization of the gel under UV light on a Gel-Doc XR Imaging System (Bio-Rad), as described [25]. Unreacted DBB, which is essentially non-fluorescent, becomes fluorescent upon cross-linking (excitation maximum 385 nm, emission, 477 nm).

### Molecular-building procedure

Structural studies on H-Ras indicate that the C-terminal segment lacks a defined structure in solution [16]. Therefore, we used a molecular-building approach for modelling this peptide. The recognition of an overall fold for the H-Ras K170-K185 peptide was based on the threading prediction from the THREADER program [40]. A three-dimensional model for the sequence of the K170-K185 peptide was built using a knowledge-based protein modelling method based on the given pair-wise target-template sequence alignment to the 156-171 spanning region of the 2.4 Å resolution X-ray structure of a CRISP family Ca^2+^-blocker (PDB code 1wvr) as template. This particular template was chosen on the basis of its high sequence identity with the Ras C-terminal peptide (sequence alignment shows a 44% sequence identity with a ratio of aligned/gap positions of 16/0 as yielded by a global alignment algorithm), its low-density map of inter-residues interactions and a relatively high relation of surface/buried residues. Nevertheless, it should be taken into account that the structure obtained is a theoretical homology model, which serves the purpose, not of defining a structure for the K170-K185 H-Ras peptide, but of illustrating the feasibility of the modifications described. The 3D-configurations of 15d-PGJ_2_ and PAO ligands were obtained from the PubChem database (http://pubchem.ncbi.nlm.nih.gov, CID codes 5280885 and 4778, respectively). Computational resources from the SWISS-MODEL homology modeling server [41] were used to achieve the final structural model for the K170-K185 peptide. The structure alignment of the 156–171 spanning region of 1wvr with the K170-K185 peptide model shows a likely identical fold, with a backbone-atoms root-mean-square deviation of 0.12 Å. The stereochemical quality of that model and its structural self-consistency were assessed with PROCHECK [42] and Verify-3D [43] programs, respectively. The initial molecular conformations for the adducts were subjected to a constrained energy minimization step in order to remove steric clashes. Computations were performed with the MacroModel 9.5 software (Schrödinger Suite 2007 Update 2, 2007). Final figures were carried out with the Swiss-Pdb Viewer v4.0 program (http://www.expasy.org/spdbv/).

### Binding of 15d-PGJ_2_-B to Ras proteins in intact cells

COS-7 cells transiently transfected with Ras plasmids were incubated with 15d-PGJ_2_-B for 2 h. Cells were lysed in 50 mM Tris-HCl, pH 7.5, 0.1 mM EDTA, 0.1 mM EGTA, 0.1 mM 2-mercaptoethanol, 0.5% SDS containing protease inhibitors (2 µg/ml each of leupeptin, aprotinin and pepstatin, and 1.3 mM ABMSF), and incorporation of 15d-PGJ_2_-B into Ras proteins was assessed by western blot with HRP-streptavidin, essentially as described [14].

### Analysis of H-Ras localization and activation

The effect of PAO on H-Ras subcellular localization was assessed by obtaining S100 and P100 fractions, as described [44]. H-Ras hydrophobicity was assessed by partition in Triton X-114, as previously described [45]. Briefly, COS-7 cells transfected with AU5-H-Ras and treated with the indicated agents were lysed in 20 mM Tris HCl, pH 7.5, 150 mM MaCl, 1% Triton X-114, containing protease inhibitors as above, for 1 h at 4°C with occasional shaking. After 3 min incubation at 37°C lysates were centrifuged at 12000 rpm for 3 min for phase separation. Aqueous phase was collected and the detergent phase was re-extracted with lysis buffer without Triton X-114. The second aqueous phase was discarded and the detergent phase was resuspended in a volume of buffer, without detergent, equal to the starting volume of lysate used for fractionation. Equal volumes of aqueous and detergent phases were analyzed by SDS-PAGE and western blot.

H-Ras activation in intact cells was monitored by two complementary assays. For a Ras activation-redistribution assay, COS-7 cells cultured on glass bottom dishes from Mattek Corp. (Ashland, MA) were transiently transfected with YFP-RBD and serum starved for 16 h, after which they were stimulated with the indicated compounds. The distribution of YFP-RBD was observed by confocal fluorescence microscopy in live cells using a Leica DMRE2 microscope (LEICA, Heidelberg, Germany). In this assay, YFP-RBD shows a diffuse distribution in non-stimulated cells, whereas Ras activation recruits YFP-RBD causing its accumulation in defined areas [8]. For quantitation, the proportion of cells showing plasma membrane-associated YFP-RBD was assessed. All experiments were repeated at least three times and representative results are shown. For a Raf RBD pull-down assay, cells were lysed in 25 mM Hepes, pH 7.5, 150 mM NaCl, 10% glycerol, 10 mM MgCl_2_, 1% NP-40, 1 mM EDTA, 0.25% deoxycholic acid containing 25 mM FNa, 1 mM sodium orthovanadate, 10 µg/ml leupeptin and aprotinin and 1.3 mM ABMSF. Cell lysates were incubated with Raf-RBD agarose beads with rotary shaking for 1 h at 4°C. After extensive washing, bound proteins were eluted by incubation for 5 min at 95°C in Laemmli sample buffer. The amount of AU5-H-Ras retained on Raf-RBD beads and that present in total cell lysates used for pull-down was assessed by western blot.

### Statistical analysis

The data shown are the mean ± SE of at least three experiments. Statistical significance was estimated with the Student's *t* test for unpaired observations. A *p* value of less than 0.05 was considered significant.
